# Spontaneous microstates related to effects of low socioeconomic status on neuroticism

**DOI:** 10.1038/s41598-020-72590-7

**Published:** 2020-09-24

**Authors:** Peifang Guo, Jinqi Cui, Yufeng Wang, Feng Zou, Xin Wu, Meng Zhang

**Affiliations:** 1 Management Institute, Xinxiang Medical University, Henan, 453003 China; 2 Department of Psychology, Xinxiang Medical University, Henan, 453003 China

**Keywords:** Electroencephalography - EEG, Cognitive neuroscience, Social neuroscience

## Abstract

Individuals with high neuroticism had the decreased control functions of anterior cingulate cortex (ACC) over amygdala (emotion regions) and low socioeconomic status (SES) had negative effects on the functions of ACC. Based on these, we hypothesized that the decreased functions of ACC might make individuals with low SES had high level of neuroticism. According to the score of objective SES (OSES) and subjective SES (SSES) scales, subjects were divided into four groups (low SSES, high SSES, low OSES and high OSES) to investigate the roles of dynamic characteristics related to the ACC in the relationships between SES and neuroticism using resting-state EEG (RS-EEG) microstates analysis. It had been found that RS-EEG microstates can be divided into four types (MS1, MS2, MS3 and MS4) and the MS3 was related cingulo-opercular brain networks (including ACC and anterior insular). As our prediction, SSES had direct effects on neuroticism relative to OSES. Moreover, the neuroticism for low SSES was positively related to the occurrence and contribution of MS3, as well as the possibilities of transitions between MS3 and MS1. Based on these, we thought that low-SSES individuals might be more difficult to inhibit the negative emotions, especially inhibit the spontaneous thoughts related to these emotions.

## Introduction

Neuroticism is an important personal trait and includes in all major modal of personality, such as three-factor^1,2^ and five-factor models^3–5^^.^ It can be operationally defined as individuals with the traits of irritability, anger, sadness, anxiety, worry, hostility, self-consciousness, and vulnerability^3,4^. Individuals with high neuroticism are often experience these negative emotions, or the circumstances evoking these negative emotions are out of proportion^6^. In addition, individuals with high neuroticism can also exist some interpersonal problems, such as self-critical, sensitive to the criticism of others, and experience greater distress in response to major life stress^7–9^. Consistent with the negative emotion perspectives, lots of studies had found that neuroticism was related to emotional brain regions such as amygdala^10–13^, the anterior cingulate cortex (ACC)^12,14^ and the medial prefrontal cortex (mPFC)^10,15–17^^.^ Moreover, the activation of amygdala was positively related to the level of neuroticism when stressful stimuli being presented^18^, while the amygdala-ACC connectivity was negatively related to the level of neuroticism^11^. Therefore, the control function of the ACC over the amygdala was decreased for individuals with high neuroticism^11^, which made them frequently experience negative emotions.

Socioeconomic status (SES) is a measurement of individual’s social and economic status relative to other people in their social hierarchy, which can be divided into objective SES (OSES) and subjective SES (SSES)^19,20^. The OSES was the traditional indicators of SES through the evaluation of income, education, and occupation of individuals’ family^21,22^ , while SSES is used to evaluate “a person’s belief about his location in a status order”^23^ and can be seen as perceived social position or subjective social status^24,25^. Considering that SSES is the cognitive average of various factors of SES, SSES is more precise evaluation of SES^20,26^. Moreover, it might be easier to make individuals with low SSES form the beliefs that their location was relatively disadvantage and the disadvantage was undeserved, which could lead to the generation of negative emotions, such as ager, resentment and dissatisfaction^27,28^. Consistent with these, Greitemeyer and Sagioglou found that SSES was a significant predictor of neuroticism and there was a negative relationship between them^29^. In addition, it had been found that low OSES and SSES had negative effects on functions of amygdala and ACC^30,31^. Due to the role of amygdala-ACC connectivity in neuroticism, low SSES might decrease the function of emotion control systems and make them be more easily develop high level neuroticism.

With the development of cognitive neuroscience, numerous studies had found that brain functions can be investigate under resting state. It had been suggested that the spontaneous brain activities played important roles in preparing functions for processing the input information^32^. Resting-state functional magnetic resonance imaging (RS-fMRI) studies furtherly determined lots of brain networks in resting sate, such as the default modal network, attentional network, salient network, visual network and so on^33–35^. Except for RS-fMRI, resting-state electroencephalogram (RS-EEG) microstates can simultaneously consider the signal from all electrodes and provide faster dynamics of these brain networks due to its high temporal resolution relative to RS-fMRI^36–39^. RS-EEG microstates is seen as “atoms of thought” and reflect the discrete mental operations related to spontaneous mental activity^40,41^. Previous studies had found that RS-EEG microstates can be divided into four classical types (MS1, MS2, MS3 and MS4), which could account for 80% variations of the EEG data^42–45^. Moreover, combing the RS-EEG microstates and RS-fMRI, Britz et al. confirmed that each one of these microstates was related to a certain brain network, namely, MS1 related to the semantic or phonological processing (superior temporal gyrus and middle temporal gyrus), MS2 related to visual and imagery processing, MS3 related to cingulo-opercular brain networks (such as ACC and anterior insular) and MS4 related to right-lateralized dorsal attentional network^43^.

According to previous studies, these dynamic characteristics of RS-EEG microstates could also reflect the function of brain networks. Specifically, duration is the time coverage of each microstate, which reflected the stability of its brain networks; occurrence is the average number of occurrences per microstate in 1 s, which represent the tendency of its brain networks being activated; contribution is the coverage rate of its brain networks in a certain time window; possibility of transition between any two microstates reflect the information flow between them^41,43,46^. Seitzman et al. furtherly found that these temporal characteristics could be altered by certain experiment conditions^47^. For example, duration, occurrence and contribution of MS4 (dorsal attention) were increased when attentional task performed; occurrence and contribution of MS2 (visual or imagery processing) were increased and duration of MS1 was decreased during eye open condition compared to eyes-closed rest; duration, occurrence and contribution of MS3 (default modal network) were decreased when attentional task performed relative to rest; the possibility of transition between MS3 and MS1 were also decreased.

According to previous studies, the control function of the ACC over the amygdala was decreased for individuals with high neuroticism and the SES had negative effects on function of the ACC. Moreover, the MS3 can be used to depict the brain networks related to ACC. Thus, we thought that the MS3 could be used to investigate the neural basis of the SES’s effects on neuroticism. In the present study, MacArthur Ladder^24^, scale of OSES^48–50^, and Neuroticism scale of the Big Five Inventory (NS-BFI) were used to measure SSES, OSES and neuroticism respectively. Based on the mean ± 1sd, the subjects were divided into low SSES, low OSES, high SSES and high OSES four groups and the RS-EEG data of them were acquired. According to previous studies, we speculated that: firstly, due to the more precise evaluation of socioeconomic status by SSES relative to OSES^20,26^, the neuroticism might be mainly influenced by SSES; secondly, the dynamic characteristics (duration, occurrence and contribution) of MS3 might be also negatively influenced by SSES; moreover, some characteristics (such as duration, occurrence, contribution) of MS3 might be related to the neuroticism for low SES.

## Method

### Ethics statement

This study was carried out in accordance with the APA requirements of human subjects with written informed consent which was in accordance with the Declaration of Helsinki. The protocol was approved by the Ethics Committee of the Xinxiang Medical University.

### Subjects

Three hundred and thirty-six right-handed subjects from Xinxiang Medical University (72% male, 28% female; mean age = 18.3, SD = 0.84) were selected to complete the measurement of the OSES and SSES scales. Then, according to the mean ± 1sd of the score of these scales, 85 subjects for SSES (45 for high SSES and 40 for low SSES) and 71 subjects for OSES (35 for low OSES and 36 for high SSES) were selected to complete the neuroticism scale of the Big five inventory and resting state EEG data collection (See Table 1). Subjects with low level of both SSES and OSSE or with high level of both SSES and OSSE were influenced by both SSES and OSSE, which might confuse the results. Thus, there were 10 subjects with low level of both SSES and OSES and 12 subjects with high level of both SSES and OSES, whose data were not included in the four groups. Subjects had no history of neurological or psychiatric disease and did not take any medication that could affect the experiment. All participants were given written informed consent to participate in the study which was approved by the ethics committee of Xinxiang Medical University.

**Table 1:** The descriptive statistics of sex, age, and neuroticism for low and high SES.

SES	Group	Sample	Sex (female/male)	Age (Mean ± SD)	Neuroticism (Mean ± SD)
SSES	Low	40	27/13	18.33 ± 0.944	37.65 ± 7.87
	High	45	35/10	18.18 ± 0.886	33.67 ± 8.32
OSES	Low	35	25/10	18.46 ± 0.817	37.54 ± 7.65
	High	36	24/12	18.00 ± 0.894	34.50 ± 8.46

### Materials

#### Subjective socioeconomic status

The SSES were measured by using the MacArthur Ladder^24,51^, in which a picture of a 10-rung ladder was presented and the subjects were asked to imagine that this ladder represents where people stand in China. They were told that at the top of the ladder are the people who are the best off—those who have the most money, the most education, and the most respected jobs, whereas at the bottom are the people who are the worst off—who have the least money, the least education, and the least respected jobs or no jobs. Subjects were asked to indicate where they think they stand at this time of their life relative to other people.

#### Objective socioeconomic status

Based on previous studies^48–50^, the family income for each month, education and occupation for mother and father were used to measure OSES. The 7-piont scale was used to rate the income: 0–600, 601–1800, 1801–3000, 3001–6000, 6001–9000 and 9001–12,000 where the unit of each number was “yuan” for RMB. The 7-piont scale was used to rate the education using the 7 items: little or no literacy, primary school, middle school, high school, junior college, undergraduate and postgraduate. The 10-piont scale was used to rate the occupation using the 10 items: unemployed or underemployed people, agricultural laborers, manufacturing workers, business and service workers, household business owners and individual industrialists and commercialists, office workers, professionals, private entrepreneurs, managers and national cadres. The higher scores for father or mother in education and occupation were used as index of each one. Then, the scores for income, education and occupation were analyzed using exploratory factor analysis to calculate the score of OSES using the factor score. The results of exploratory factor showed that the one factor can explain 59.84% of the total variance. The score of OSES was calculated using the formula: OSES = 0.815 × Z_income_ + 0.599 × Z_education_ + 0.879 × Z_occupation_, where the number was the score of each factor, the “Z” was the Z score of each factor and the higher of the score, the higher OSES.

#### Neuroticism scale

The neuroticism was measured using the neuroticism scale of the Big Five Inventory (NS-BFI). The NS-BFI was composed of 12 items and subjects were asked to be rated on a 5-point scale ranging from 1 (very disagreement) to 5 (agreement). After the score of 4 items being reversed, the total score of NS-BFI were computed and the mean score were 35.541 ± 8.306 with the score ranging from 16 to 56. The reliability of NS-BFI was 0.831 in our sample.

### Resting-state EEG data acquisition

The RS-EEG recording was about 6 min, in which subjects were asked to relax, keep their eyes open, focus on the “+” that appeared in the center of the screen, and not to move their body or head. Brain electrical activity was recorded from 64 Ag–AgCl scalp sites according to the international 10–20 system in an elastic cap (Neuro Scan Product). During recording, all electrodes were referenced to Cz and re-referenced off-line to linked mastoids. Channels for horizontal and vertical EOG were computed offline from electrodes recorded from the outer canthi of the eyes and from above and below the right eye, respectively. Electrode impedance was kept below 5 kΩ. EEG was sampled on-line with a frequency of 500 Hz DC-amplifiers with a band-pass filter of 0.1–100 Hz.

### RS-EEG microstate preprocessing

The EEG data was preprocessed using EEGLAB (https://sccn.ucsd.edu/eeglab/index.php) in MATLAB 2018b (https://cn.mathworks.com/). Data was filtered off-line by a band-pass filter of 2–20 Hz and an independent component analysis (ICA) was used to reject the components related to the blinks, eye movements, eyes drift, head movement, power-line interference or electro cardio. The artifact free data was recomputed against the average, according to previous studies^42,46,52,53^ and was average re-referenced. Then the data was segmented into 180 epochs with epoch length of 2000 ms. EEG epochs with amplitude values exceeding ± 80 μV which might be contaminated by strong muscle artifacts were also rejected.

**Figure 1:**
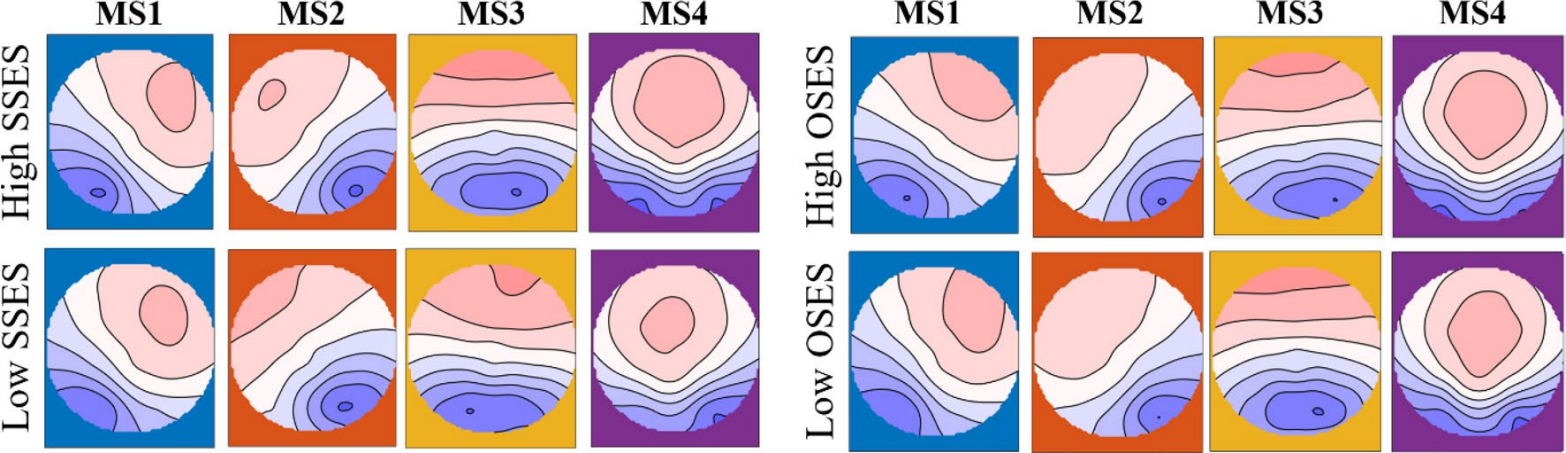
The four microstate topographic maps (MS1, MS2, MS3, MS4) for low SSES, high SES, low OSES and high OSES.

The RS-EEG microstates were calculated as following according to previous studies^42,46,52,53^. First, the global field power (GFP) which was defined as the EEG potential variance across all electrodes and only the topographies at peaks of GFP were further analyzed. After that, the Atomize-Agglomerate Hierarchical cluster (AAHC) were performed to analyze the microstates with the polarity of each topographical map being disregarded. The AAHC was a modified k-means which was devised specifically for microstate and can provide unique clusters^54^. Thirdly, a cross-validation criterion was used to identify the optimal number of clusters, which was the best solution to find the minimal number of template maps with maximal global explained variance^43,55^. According to our data, four clusters maps (MS1, MS2, MS3, MS4) for low SSES, high SSES, low OSES and high OSES were found respectively (See Fig. 1), which same as previous studies^42,43,46,53^. Then, these maps for each group was used to fit their original data based on the global map dissimilarity (GMD) and each time point was labeled with one cluster map it correlated to best^56^. Finally, the dynamic characteristics of each topographic map was calculated using the marked data, namely the mean duration of each microstate (duration), the mean frequency of each microstate (occurrence), and the mean coverage rate of each microstate in the epochs (contribution)^41,43,46^.

### Statistical analysis

#### Microstate parameters

The sex and age were controlled as covariates in the following analysis. In the RS-EEG microstate, the MS3 was selected as interest of RS-EEG microstate and the 2 (SES: SSES vs OSES) × 2 (SES level: low vs high) two-way between-subject ANOVA was used to analyze the effects of SES on parameters (duration, occurrence and contribution) of MS3 respectively. The significant threshold for F test was *p* < 0.05 with Bonferroni correction (*p* = 0.017 which is the 0.05 divided by the number of the parameters). Then, the relationships between the significant parameters and neuroticism were analyzed using Pearson correlation for low SSES, high SSES, low OSES and high OSES respectively. According to our results, occurrence and contribution were significant among low SSES, high SSES, low OSES and high OSES. Thus, the significant threshold for Pearson correlation test was *p* < 0.05 with Bonferroni correction (*p* = 0.025 which is the 0.05 divided by the number of the parameters).

The transitions between MS3 and other RS-EEG microstates (MS1, MS2, and MS4) were also selected to analyze the effect of RS-EEG microstates on neuroticism using Pearson correlation for low SSES, high SSES, low OSES and high OSES respectively. To explore the relationships between neuroticism and other temporal characteristics of MS3, a conservative significant threshold (*p* = 0.002) with Bonferroni correction were used, which is the 0.05 divided by 24 [2 (the number of SES) × 2 (the number of SES level) × 6 (the number of the transitions)].

## Results

### Behavioral results

Total score of NS-BFI for low SSES (37.65 ± 7.87) were higher than that for high SSES (33.67 ± 8.31) [t (83) = 2.26, *p* = 0.026] (See Table 1). Total score of NS-BFI for low OSES (37.54 ± 7.65) were not significantly different from that for high SSES (34.50 ± 8.46) [t (69) = 1.59, *p* = 0.117] (See Table 1). Moreover, the total score of NS-BFI were conformed to normal distribution according to Shapiro–Wilk test (for low SSES *p* = 0.475; for high SSES *p* = 0.593; for low SSES *p* = 0.277; for high SSES *p* = 0.903).

### Effects of SSES on RS-EEG microstates

The ANOVA results showed that the interactions of SES × SES level were significant for occurrence of MS3 [F (1, 150) = 8.629, *p* = 0.004, η^2^ = 0.055] and marginally significant for contribution of MS3 [F (1, 150) = 5.637, *p* = 0.019, η^2^ = 0.036], while there are no other significant results; the main effects of SES and SES level, as well as the interaction of SES × SES level were not significant for duration of MS3 (See Table 2).

**Table 2:** The results of two-way ANOVA analyzing the data of duration, occurrence and contribution.

	Duration			Occurrence			Contribution		
	F	*p*	η2	F	*p*	η2	F	*p*	η2
SES	0.394	0.531	0.003	5.760	0.018	0.037	2.755	0.099	0.018
SES level	1.764	0.186	0.012	0.924	0.338	0.006	2.232	0.137	0.015
SES*SES level	1.707	0.193	0.011	**8.692**	**0.004**	**0.055**	***5.637***	***0.019***	***0.036***

The bold representing the significant results at *p* < 0.05 with Bonferroni correction.

The bold and italics representing the marginally significant results at *p* < 0.05 with Bonferroni correction.

**Figure 2:**
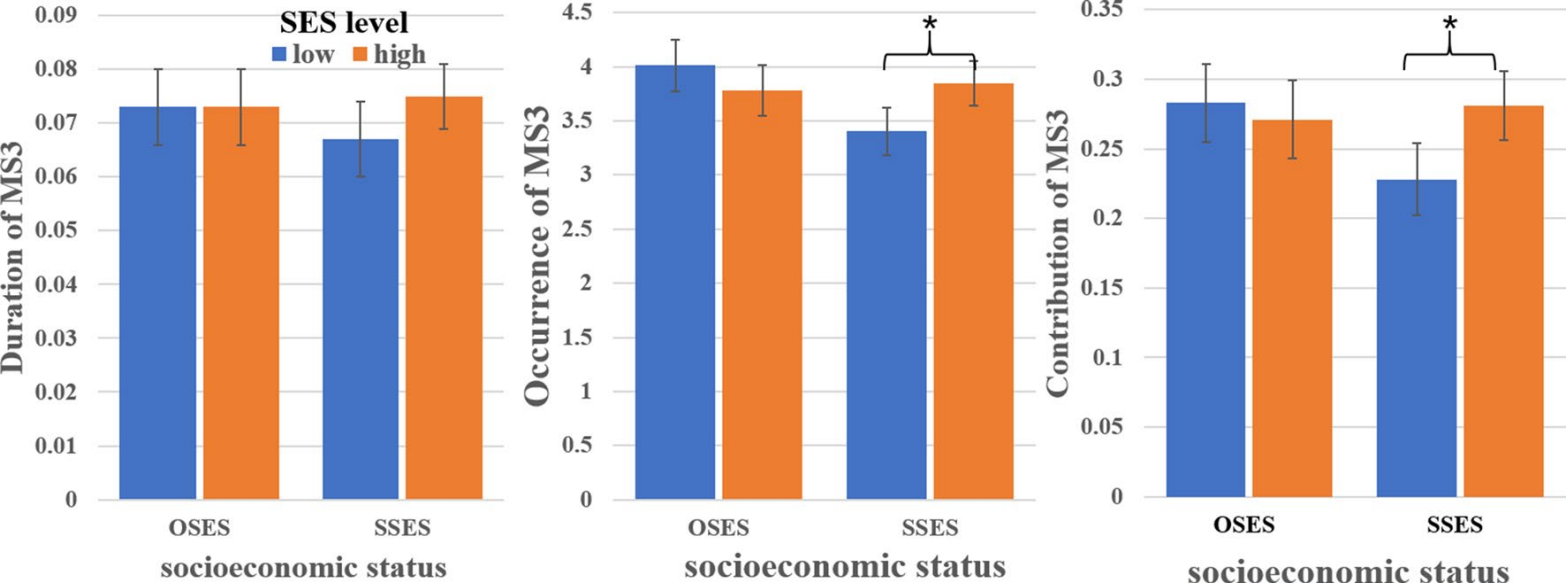
The results of the differences between in duration, occurrence and contribution of MS3 for low SSES, high SES, low OSES and high OSES.

The simple effect showed that contribution of MS3 for low SSES were lower than high SSES [F (1, 150) = 8.303, *p* = 0.005, η^2^ = 0.052], while there was no significant difference between low and high OSES [F (1, 150) = 0.343, *p* = 0.559, η^2^ = 0.002]; occurrence of MS3 for low SSES were lower than high SSES [F (1, 150) = 8.466, *p* = 0.004, η^2^ = 0.053], while there was no significant difference between low and high OSES [F (1, 150) = 1.773, *p* = 0.185, η^2^ = 0.012] (See Table 3 and Fig. 2).

**Table 3:** The descriptive statistics of the parameters (duration, occurrence and contribution) for MS3.

SES	Duration	Occurrence	Contribution
Low SSES	0.067 ± 0.017	3.405 ± 0.668	0.228 ± 0.086
High SSES	0.075 ± 0.023	3.851 ± 0.699	0.281 ± 0.090
Low OSES	0.073 ± 0.021	4.029 ± 0.604	0.285 ± 0.075
High OSES	0.073 ± 0.016	3.758 ± 0.829	0.268 ± 0.081

### The relationships between neuroticism and microstates for low and high SSES

The results showed that the contribution of MS3 were positively related to total score of NS-BFI for low SSES (r = 0.432, *p* = 0.007). In addition, results of the transitions between MS3 and other microstates showed that the possibility of transitions from MS1 to MS3 (r = 0.479, *p* = 0.002) and from MS3 to MS1 (r = 0.519, *p* = 0.001) were both positively related to total score of NS-BFI for low SSES; there are no other significant results (See Table 4).

**Table 4:** The relationships between neuroticism and parameters of RS-EEG microstates.

	OSES		SSES	
	Low	High	Low	High
**Occurrence**				
MS1	0.145 (0.421)	− 0.081 (0.648)	− 0.080 (0.631)	− 0.129 (0.411)
MS3	− 0.021 (0.908)	− 0.269 (0.124)	0.266 (0.107)	− 0.119 (0.446)
**Contribution**				
MS1	0.217 (0.224)	− 0.062 (0.728)	− 0.038 (0.821)	− 0.062 (0.695)
MS3	0.154 (0.392)	− 0.268 (0.125)	**0.432 (0.007)**	− 0.018 (0.909)
**Transition**				
MS1 to MS3	0.309 (0.080)	− 0.274 (0.116)	**0.479 (0.002)**	− 0.132 (0.398)
MS2 to MS3	− 0.218 (0.223)	− 0.019 (0.917)	0.260 (0.115)	− 0.014 (0.930)
MS3 to MS1	0.347 (0.048)	− 0.263 (0.133)	**0.519 (0.001)**	− 0.135 (0.390)
MS3 to MS2	− 0.224 (0.211)	− 0.059 (0.739)	0.207 (0.212)	− 0.035 (0.821)
MS3 to MS4	0.133 (0.461)	− 0.173 (0.329)	0.045 (0.790)	0.107 (0.496)
MS4 to MS3	0.183 (0.309)	− 0.216 (0.219)	− 0.007 (0.969)	0.069 (0.661)

The bold representing significant results with *p* < 0.05 with Bonferroni correction.

## Discussion

In present study, the negative effects of low SES on neuroticism and its neural basis were investigated by using the RS-EEG microstate analysis. According to previous studies, we thought that the temporal characteristics of MS3 (related to ACC) might be negatively influenced by lower SSES, which might play important roles in higher neuroticism for low SSES. As our prediction, the results showed that both occurrence and contribution of MS3 were decreased for low SSES relative to high SSES, but this relationship was not observed through comparing low and high OSES. More interesting, the contribution of MS3 and the transitions between MS3 and MS1 were positively related to neuroticism.

Firstly, according to our results, the SSES had more direct effect on neuroticism relative to OSES, which found that the low SSES had more negative effects on neuroticism. Moreover, the occurrence and contribution of MS3 were decreased for low SSES relative to high SSES. Some previous studies found that the low socioeconomic status might make individuals exposure to stress^30,57^. Evidences from animals and humans demonstrated that stress had detrimental effects on emotion, socio-emotional processing, and cognitive control related brain regions, such as amygdala^58,59^ and ACC^58,60^. According to previous study, the MS3 were related to positive BOLD activation of ACC, medial prefrontal cortex (mPFC), bilateral inferior frontal gyrus (IFG) and right amygdala^42^. Thus, the negative effects of low SSES on occurrence and contribution of MS3 might reflect that the emotional network was influenced by SSES. Moreover, it had been found that ACC/mPFC played important role in suppression of the activation of amygdala when individuals be confronted with threat stimuli^61–66^. Therefore, lower frequency of MS3 for low SSES than high SSES might reflect that low SSES was difficult to inhibit the regions of generating negative emotions.

Secondly, the positive relationship between neuroticism and contribution of MS3 for low SSES was found in this study. It had been suggested that the individuals with high neuroticism had the traits of irritability, anger, sadness, anxiety, worry, hostility, self-consciousness, and vulnerability^3,4^ and more frequently experience negative emotions^6^. Moreover, as Khanna et al. reviewed, the occurrence might reflect the tendency of neural generators being activated for certain RS-EEG microstate, while the contribution might reflect neural generators for certain microstate having more time coverage relative to other microstates^41^. From this perspective, the positive effect relationship between neuroticism and contribution of MS3 might reflect that low SSES had negative effects on the efficacy of the control function of ACC to negative emotions. In other word, the longer time activity of ACC was required for individuals with low SSES in unit time to control the negative emotions.

Thirdly, we also found that the neuroticism was positively related to the possibilities of transitions between MS1 and MS3. Britz et al. found that MS1 was related to the negative BOLD activation of linguistic brain regions, such as bilateral superior and middle temporal gyrus as well as the left middle frontal gyrus^43^. It seems amazing that the emotional and linguistic networks had the relationship of sequential activation, but it might be reasonable to concern the role of rumination in negative emotion. Rumination is a pattern of repetitive thoughts related to negative emotions, such as depression and anxiety^62–64^, and is also seen as maladaptive self-reflection on these negative thoughts^65^. Thus, rumination is related to negative thinking and make individuals ruminate spontaneously talk about troubling problems^66–68^. On these perspectives, the sequential activations between MS1 and MS3 for low SSES might reflect that with the neuroticism increasing, low-SSES individuals might be more difficult to inhibit the spontaneous thought related negative emotions.

In summary, the rapidly changing characteristics of spontaneous brain activities related to the negative effects of low SSES on neuroticism were investigated by RS-EEG microstates analysis. The results showed that the low SSES had detrimental effects on occurrence and contribution of MS3. Moreover, contribution of MS3 and the possibilities of transitions between MS1 and MS3 were positively related to neuroticism for individuals with low SSES. According to these results we thought that with the level of neuroticism increasing, individuals with low SSES were more difficult to inhibit the negative emotions, especially inhibit the spontaneous thoughts related negative emotions.

## Limitations and future directions

Although lots of interesting results being found, there were some limitation should be concerned. Firstly, it had been found that neuroticism might be influenced by some other traits and state factors, such as self-esteem^69^, trait anxiety^70,71^, depression^72,73^. Although sex and age were controlled in present study, these potential personal factors were not controlled. Thus, these factors should be controlled to furtherly investigate the relationship between SES and neuroticism and its neural basis in future study. In addition, as our results showed, SSES had more direct effects on neuroticism relative to OSES. However, due to the samples shared low SSES and OSES or high SSES and OSES being too small, we did not know whether the results through comparing low (SSES and OSES) and high (high SSES and OSES) SES were the same to the results in this study. Thus, much bigger sample should be used to investigate these effects. Thirdly, considering that only college students were employed in this study, which might be cautious to explain other groups with different ages (such as children, old adult and so on). Thus, other subjects with different ages should be select to investigate the relationships between SES and neuroticism in the future study. Finally, considering that the MS3 were related to ACC, medial prefrontal cortex, bilateral inferior frontal gyrus and right amygdala^43^, we could not confirm which regions was more serious influenced by SSES and the roles of them in neuroticism. The neural basis of the relationships between SES and neuroticism should be furtherly studied using more spatially accurate technique (such as fMRI).
